# Comparative Study of Machine Learning Models for Bee Colony Acoustic Pattern Classification on Low Computational Resources

**DOI:** 10.3390/s23010460

**Published:** 2023-01-01

**Authors:** Antonio Robles-Guerrero, Tonatiuh Saucedo-Anaya, Carlos A. Guerrero-Mendez, Salvador Gómez-Jiménez, David J. Navarro-Solís

**Affiliations:** 1 Unidad Académica de Ingeniería I, Universidad Autónoma de Zacatecas, Zacatecas 98000, Mexico; 2 Unidad Académica de Ciencia y Tecnología de la Luz y la Materia, Universidad Autónoma de Zacatecas, Zacatecas 98047, Mexico

**Keywords:** precision beekeeping, bee acoustics, beehive monitoring, queenless state

## Abstract

In precision beekeeping, the automatic recognition of colony states to assess the health status of bee colonies with dedicated hardware is an important challenge for researchers, and the use of machine learning (ML) models to predict acoustic patterns has increased attention. In this work, five classification ML algorithms were compared to find a model with the best performance and the lowest computational cost for identifying colony states by analyzing acoustic patterns. Several metrics were computed to evaluate the performance of the models, and the code execution time was measured (in the training and testing process) as a CPU usage measure. Furthermore, a simple and efficient methodology for dataset prepossessing is presented; this allows the possibility to train and test the models in very short times on limited resources hardware, such as the Raspberry Pi computer, moreover, achieving a high classification performance (above 95%) in all the ML models. The aim is to reduce power consumption and improves the battery life on a monitor system for automatic recognition of bee colony states.

## 1. Introduction

Technological tools, such as monitoring systems, have been developed to capture, analyze and understand the parameters of bee colonies in order to reduce mortality and improve traditional apiculture; this is the aim of precision beekeeping [1]. A parameter of particular interest for researchers is the acoustics of bee colonies; it can be effectively analyzed to understand and predict critical states of bee colonies [2,3]. Some examples of the acoustics patterns present in bees colonies include the process of swarming, a bee colony changes its normal activity and produces a specific buzz before the queen leaves the colony with part of the swarm [4,5]. On the other side, a bee colony produces a characteristic sound when the queen is absent, the queenless state causes stress in the colony members, and the usual activities become chaos. Furthermore, the high temperature inside the hive is another factor that can affect the sound of a colony [6,7,8]; the bees placed at the entrance of the colony flap their wings to ventilate and reduce the temperature that can be mortal for the brood nest. Finally, when predators threaten bees, the entire colony produces a characteristic sound as a defensive behavior [9]. To capture and analyze the bee colony acoustics, several researchers have been interested in developing monitoring systems based on low resources computers such as Raspberry Pi (RPi) [8,10,11,12,13,14,15,16,17,18,19,20,21]. The main goal is to identify colony states automatically by using these technologies and reduce invasive inspections that cause stress in colony members and reduction of the productivity.

The main objective of this work is to compare the most used ML models for acoustic pattern classification in bee colonies and find a solution with a balance between performance and low consumption of computational resources. Classical ML methodologies are simple, fast, and easy to train. The main reason to implement these methodologies is that most require less computational power than deep learning techniques, which usually are more complex and time-consuming and can be limiting factors for real-time applications [22]. Furthermore, classical ML architectures can be easily implemented in a platform with limited computational resources, such as RPi, whose principal advantage is its support and availability. Moreover, the use of Python and its open-source libraries, such as *scikit-learn*, allows the fast and easy development of classifiers based on ML algorithms. An important challenge in developing intelligent systems for the automatic recognition of bee colony states is the lack of computational resources and battery life. In a real-life scenario, most apiaries are in remote places where access to electric energy is not guaranteed. Therefore, a monitoring system must be powered by solar energy, and cloudy days can be a problem, even if the primary system is a single-board computer [23]. Among the most critical tasks, a recognition system must perform include sound recording, information storage, feature extraction, and pattern classification. An alternative to reduce power consumption and increase the battery life of a monitoring system is decreasing CPU usage. The proper ML model selection can improve battery life; for that reason, five ML models were compared: Logistic Regression (LR), Support Vector Machines (SVM), Random Forest (RF), K-Nearest Neighbors (KNN), and Neural Networks (NN), the aim was to evaluate the computational requirements by comparing the execution time of the ML models on an RPi 3. Furthermore, several metrics were computed to provide a complete overview of the models’ performance: confusion matrix, accuracy, precision, recall, F1-score, and ROC (receiver operating characteristic) curves. Furthermore, a simple prepossessing step allows training the models on limited-resources hardware.

The rest of the paper is organized as follows: Section 2 shows a literature study on monitoring systems and ML models for recognition of colony states, in Section 3 the methodologies for sound classification, data prepossessing, and characteristics about the dataset are described. Then, results and discussion are presented in Section 4. Finally, Section 5 provides the conclusions and future work.

## 2. State of the Art

Several studies suggest that ML algorithms and classical methodologies for speech recognition can be effectively adapted for acoustic pattern classification in bee colonies; such methodologies are being implemented effectively to identify bee colony states achieving high correct classification rates. Furthermore, CNN architectures have proven effective for audio classification and have been used for bee acoustic pattern recognition. The following paragraphs review the most important research on identifying acoustics patterns in precision beekeeping.

SVM is among the most used ML techniques for the identification of the health status of bee colonies by analysis of acoustic patterns. Amro et al. [19] implemented two ML models, an SVM model and a linear discriminant analysis, to determine the infestation level of beehives due to varroa mites. The colony’s acoustics were recorded using a credit card-sized computer and electret microphones; the prepossessing and recognition tasks took place in the monitoring system. A pattern of an infected colony is compared with a healthy colony, and both methods can successfully be used to identify differences between healthy and infected beehives.

Nolasco et al. [24] compared two methodologies for bee sound identification, a convolutional neural network (CNN) and an SVM model. The dataset is a selection of sounds of beehives recorded in various conditions of two projects, Open Source Beehive (OSBH) and NU-Hive. The tested models have the possibility to identify bee sounds from external sounds such as traffic or birds. Mel Frequency Cepstral Coefficients (MFCCs) and Mel spectra were used for feature extraction. Furthermore, the signals were split into segments of different sizes to analyze the performances. In this study, the SVM model performed better than the CNN. However, a special issue is the possibility of generalizing on unseen colonies.

In the classification of bee sounds and external sounds, Kim et al. [25] compared conventional ML models: RF, SVM and extreme gradient boosting with two CNN architectures: VGG-13 and Shallow CNN. The dataset consists of the sound of the OSBH project; the outputs were labeled as bee and nobee. They used Mel spectrograms, MFCCs, and constant-Q transform for feature extraction. The results show that VGG-13 has the best performance, achieving 91% of accuracy.

To discriminate swarming activity from regular activity in bee colonies, Zgank [26] implemented a model based on Hidden Markov Models (HMM), widely used for human speech recognition, and MFCCs for feature extraction. The dataset consists of acoustic patterns of beehives from an open-source project. The model can achieve an accuracy of 80% in the classification of swarm bee activity. Zgank [27] improved the study and compared the performance of HMM and Gaussian Mixture Models in the classification of the acoustics of bee colonies. In addition, Zgank compared MFCCs and linear predictive coding as feature extraction methods in this work. Several metrics were provided to reflect the performance, and the highest accuracy was achieved with HMM model and MFCCs features. In another work, Zgank [28] improved previous results by using deep neural networks and MFCCs. A comparison between ML models to detect swarming and non-swarming activity was made by Dimitrios et al. [29]. In the study, they compare the performance of KNN, SVM and U-Net CNN, they combine the acoustic samples with measures of the temperature inside the hive, and humidity and temperature outside the hive. The signals were filtered with a low pass filter. In the pre-processing step, they extract the Fast Fourier Transform (FFT) of the signals, considering the range of frequencies of 150–600 Hz. The results reveal that SVM achieves a better performance than CNN.

For the detection of queenless bee colonies, Nolasco et al. [30] implemented a classification methodology based on previous works, SVM and a CNN models were implemented for the task. The dataset consists of the beehive sound of the NU-Hive project. They proposed MFCCs, Mel spectrograms, and the Hilbert Huang Transform (HHT) for feature extraction. According to previous results, their SVM model performs better than CNN using HHT and MFCCs. The critical point is that a better performance can be achieved when an appropriate feature extraction procedure is used. Howard et al. [31] implemented a different approach to classify queenless states in bee colonies by using self-organizing maps; for feature extraction, they applied power spectral density and S-Transform. However, the results show that the model cannot classify the beehive state; however, the problem can be related to the dataset’s characteristics. Another study to detect queen presence was made by Cejrowski et al. [32]. A series of experiments were conducted to reproduce the queen’s absence. This work implemented linear predictive coding as feature extraction and as learning algorithm SVM; the classification algorithm can identify the acoustic patterns of healthy and queenless colonies. To improve the detection of queenless states by CNN, Orloswska et al. [33] propose a simple transformation to increase the classification performance. The datasets consist of audio data from the OSBH and NU-Hive projects. The transformation is applied to the spectrograms and consists of two-steps dimension reduction. The authors claim that this transformation represents a better generalization, and the CNN achieves an accuracy of 96%. Another approach to improve the identification of queenless states is presented by Peng et al. [34]; this work proposed a Wiener filter to reduce the signal-to-noise ratio. After that, MFCCs methodology was applied for feature extraction, and the dataset was used to train a Multi-layer perceptron neural network. The authors conclude that the filter increases the classification accuracy by 12% compared with the non-filtered signal. Following the topic of queenless state identification of bee colonies, Robles et al. [35,36] designed a methodology for bee acoustic classification based on LR and MFCCs for feature extraction. A monitor system based on RPi 2 and omnidirectional microphones was implemented for the acoustic recording. An acoustic pattern of a healthy colony was compared with a pattern of queenless colonies. By using this methodology, the patterns were correctly identified with a high correct classification rate (above 90%).

When bees are exposed to chemicals, they respond with no natural sound. Zhao et al. [10] recorded the sounds of bees exposed to compounds of acetone, trichloromethane, glutaric dialdehyde, and ethyl ether by using microphones and an RPi 3 model B. For feature extraction, they used the MFCCs methodology. They implemented three classification models: KNN, SVM and RF; also, they performed a principal component analysis to select the most relevant coefficients. The algorithm with the best results is SVM, and is able to identify when the bees are exposed to specific compounds of acetone. Another research to detect the acoustic response of beehives exposed to trichloromethane is presented by Sharif et al. [37]; this study proposes a methodology known as Soundscape indices for feature extraction and it was compared with MFCCs methodology. An RF model was trained to compare the feature extraction methodologies, and the soundscape indices achieved a better performance.

A comparative of standard ML techniques (LR, KNN, SVM, and RF) and CNN for the classification of audio samples of bee colonies and ambient noise was performed by Kulyukin et al. [15]. The audio samples were recorded by microphones and an RPi computer. The study analyzed two datasets; the first consists of 10,260 audio samples, and the second of 12,914 audio samples. For feature extraction, a methodology based on MFCCs was used. Moreover, the study performs an audio classification experiment on an RPi 3, and the results show that better performance was achieved with deep learning techniques.

SVM and MFCCs features have been used to identify circadian rhythm in bee colonies by Cejrowski et al. [38], this work aims to classify bee sound activity in days and nights and the period when the activity is the lowest over the day. The monitor system consists of an RPi computer and an analog microphone; the sound samples were recorded at a sample rate of 3 kHz and 12-bit resolution in intervals of 15 min. The colony’s lowest activity period was found from 11 pm to 4 am.

ML models have been used to discriminate bee species by analyzing the flight sounds of bees and hornets [39]; the sound of three species of bees and one species of hornet was recorded. They use an SVM model and MFCCs for feature extraction to classify the sound samples. The model can accurately discriminate between flight and environmental sounds (such as bird and background noise).

## 3. Materials and Methods

### 3.1. Dataset Description

For this study, the acoustic samples were recorded from five colonies of Carniola honeybee (Apis Mellifera Carnica) selected from an apiary in Zacatecas, México. The characteristics of the chosen bee colonies were different, two healthy queenright colonies with a vast population (approximately 60 thousand bees), two queenright colonies with a medium population (40 thousand bees), and a low-population queenless colony (30 thousand bees). This last colony was found in that condition. An expert beekeeper evaluated the colonies’ size and health status based on the characteristics and number of bees on the hive frames. The queenless condition was artificially created; after monitoring the colonies for 15 days, no significant changes were found in the acoustic patterns when the samples were analyzed by singular value decomposition; a detailed description of the study can be found at [35]. After that, two queens were removed, one from a healthy colony and one from a medium-population colony. Table 1 summarizes the information on the bee colonies.

**Figure 1:**
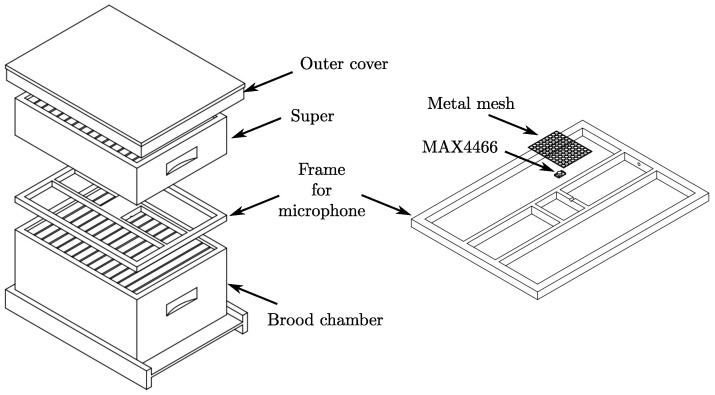
Frame design and microphone placement in the hive, on the left, and protected microphones with metallic mesh against wax on the right.

**Figure 2:**
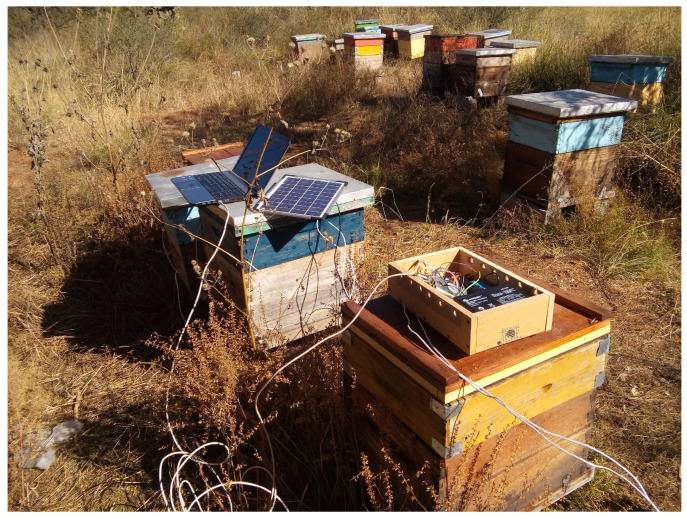
Monitor system setup, installation of the microphones in five bee colonies of the apiary located in Zacatecas, México.

The colonies were monitored from 15 March through 30 April in 2018. The acoustic patterns were recorded with a monitor system based on an RPi 2 and omnidirectional electret microphones (model MAX4466), with a frequency range of 20–20K Hz and adjustable gain. A frame was designed to match the hive’s external dimension and hold up the microphones. The frame was placed over the brood chamber, under the outer cover in colonies without supers; in colonies with supers, the microphone was placed between the brood chamber and the first super, Figure 1. The microphones were protected with metallic mesh to prevent being covered with wax; every week, the mesh was cleaned to remove wax remnants and avoid sound obstruction. A dsPIC33EP512MC controller was used as a signal digitizer. The data were sent from the dsPIC microcontroller to the RPi 2 via SPI (Serial Peripheral Interface) bus and finally stored into a micro SD memory card. The monitor system was powered by a 10 ah power bank and a 10 W solar panel. Figure 2 shows the implementation of the monitor system in the beehives. The sampling frequency was set to 4 kHz; most of the acoustic in a bee colony is in the range of 100–500 Hz [40,41,42,43]; therefore, the sampling frequency satisfies the Nyquist–Shannon sampling theorem. Every sample is 30 s long and were recorded in 10 min intervals. The pre-processing and feature extraction steps were carried out on a desktop PC.

### 3.2. Feature Extraction and Dataset Prepossessing

For feature extraction, a classical MFCCs methodology was implemented. MFCCs has been effectively used in the preprocessing step of acoustic patterns of bee colonies [10,15,24,30,35,36]. The classical methodology includes the following stages: a pre-emphasis filter, windowing of the signal, application of the fast Fourier transform, warping the frequencies on a Mel scale, the application of a triangular filter bank, and the inverse discrete cosine transform. The MFCCs were computed in MATLAB with the function *mfcc* [44] using the parameters shown in Table 2. For this work, only the first 12 Mel coefficients were computed, and the first and second-order derivatives were not included. After the feature extraction procedure, the mean value of MFCCs was calculated to reduce the dataset size; this step reduces the time required to train the models, allowing the possibility to be executed in low resources computers. Finally, the data were standardized by using z-scores. The dataset consists of 12 features and a total of 720 instances or samples, 144 for each colony; the outputs of the dataset are queenright colonies (QR), queenless colonies (QL), and low population queenless colonies (LPQL). Every feature in the model was labeled with *mean* and the number corresponding to the Mel coefficient.

### 3.3. ML Models

The model known as SVM is considered one the most flexible and efficient machine learning models [45]. SVM is a method that uses a kernel function to map the data into a high-dimensional feature space; a support vector defines a hyperplane to find the maximum separation between two binary classes. New data can be classified depending on the relative position of the hyperplane. The KNN model has been used widely used for classification tasks for its simplicity, effectiveness, and robustness. KNN predicts new samples based on the K-nearest neighbors on the training set. In KNN, a given training set data are defined by n-attributes and plotted in a high dimensional space where each axis corresponds to an attribute. For new data classification, KNN searches the sets of data, closest to similar. The similarity is a measure of distance between two instances and is typically computed by standard Euclidean distance. LR is one of the simplest models, and its prediction equation is easy to implement. LR is frequently used for binary classification; however, it can be used for multiclass classification. First, the model aims to find a boundary among classes. Then, the model computes a series of predictor parameters based on the features and the outcomes. The LR model response is the probability of belonging to a particular class, and the values are mapped to a value between 0 and 1 via an S-shaped logistic function. For classification, a cutoff value must be set, typically 0.5. RF is an ensemble method that consists of a large number of decision trees from random subsets of data. Each uncorrelated decision tree in the RF produces a result; in classification, to predict the new class, the trees with the most votes become the prediction model [46]. An advantage of RF is that it can handle multiclass classification [47]. The NN model is inspired by how the brain works [48]; it was proposed as a simplified model of biological NN. An NN consists of an input data layer, an output layer, and hidden layers between those. The input layer receives the information to train the model, every layer in the NN is composed of interconnected nodes, the input data are processed through the nodes by multiplying for a weight and a training algorithm, and finally, in the output layer, the solution of the problem is processed.

### 3.4. Performance Evaluation

The problem under analysis involves a multiclass classification of three outputs: healthy queenright colonies, queenless colonies where the queens were removed, and a queenless colony with a low population. In addition, several metrics were computed to assess the predictive performance of the ML models and provide a complete overview: confusion matrix, accuracy, precision, recall, F1-score, and the area under the ROC curve.

The Confusion Matrix (CM) is a table that depicts the correct and incorrect predictions of a classification model in labels as true positive (TP), false positive (FP), true negative (TN), and false negative (FN). CM provides a straightforward way to visualize the performance of the models; several metrics are derived from CM. *Accuracy* is the fraction of correctly classified samples from all the samples. *Precision* is the portion of samples predicted as positive that were correctly classified as positive. *Recall*, also known as sensitivity, is the fraction of all positive samples that were correctly classified as positive. *F1-score* is the combination of Precision and Recall, which is the harmonic mean of both metrics, and *Misclassification rate*, is the fraction of samples that were incorrectly classified. The formulas are listed in the summary Table 3. In the case of multiclass classification, the labels of CM must be reassigned for each class.

The area under the ROC curve is another performance measure and is a method that combines sensitivity and specificity into a single value. ROC curve is a plot created by evaluating the class probabilities of a model across a continuum threshold [45]. For each threshold, the resulting true positive rate (sensitivity) and the false positive rate (specificity) are plotted against each other. For example, an optimal classifier would produce a curve in the upper left corner, and the area under the ROC curve of the model would be 1; on the other hand, a value of area under the ROC curve below 0.5 represents a model that is worse than chance [49]. We will refer to AUC as the area under the ROC curve. ROC curves are usually used in binary classification problems; however, their use can be extended to analyze multiclass problems, binarizing the outputs of the problem. The technique known as “one-vs-all” (OvA) or “one-vs-the rest” is used to solve the multiclass classification problem, in this case, to generate the ROC curves. The OvA technique has proved to be an efficient and straightforward way to deal with multiclass classification tasks [50]; the technique involves the training of a binary classification per class as positive and the rest outputs as negative. Then the process is repeated for each output.

For validation, a k-folds cross-validation procedure was used; this methodology consists of partitioning the dataset into k sets or subsets; the model is trained using all the samples except by the first subset, and the held-out subset is used as a test sample to estimate the model performance. The subset held out is returned, and the procedure is repeated by extracting a second subset. Finally, the k estimations are usually summarized with the mean value. Given the dataset size (720 samples) an eight-fold cross-validation was implemented.

The SVM and LR models were initially developed for binary classification tasks; in order to deal with multiclass problems, the ML models are analyzed under the OvA schema by the *scikit-learn* library. On the other hand, NN, RF, and KNN models can support native multiclass classification tasks; however, RF can achieve higher prediction performance when the problems are analyzed with the OvA scheme [51].

Finally, another performance measure of particular interest in this work is the execution time of the training and testing process of the ML models on the RPi 3. Most of the time, the main task of a monitoring system will be the classification of new samples and is the most important time measure; however, if the training time is relatively fast, the training phase can take place on the monitor system. The specifications of the RPi 3 and the setup characteristics are shown in Table 4. The ML codes were written by using the library *scikit-learn* [52] version 0.23.1 on Python 3.7. Finally, the hyperparameters (the specific values that control the learning process) for each ML model were found by process of random search [53], which is a practical and efficient methodology to find the optimal configuration of the ML models. The name of the function of every ML model and hyperparameter are shown in Table 5. The hyperparameters were found by using the function *RandomizedSearchCV* from the library *scikit-learn*.

## 4. Results and Discussion

### 4.1. Sensitivity Analysis

**Figure 3:**
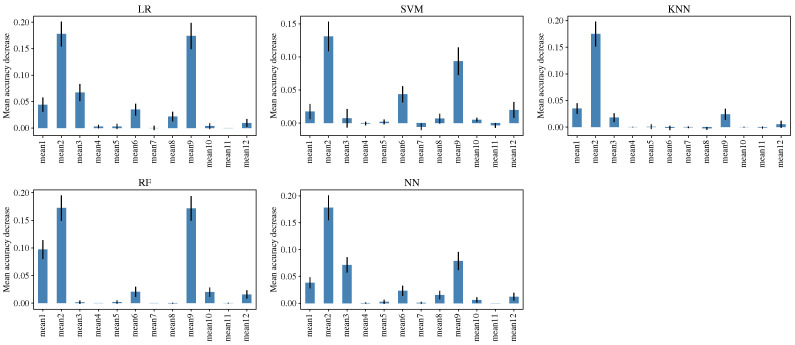
Sensitivity analysis to examine the feature importance for each ML model.

A sensitivity analysis was conducted to examine the impact of each feature on the performance of the models. The analysis was performed through the permutation of the feature values by using the function *permutation_importance* of the *scikit-learn* library. The feature importance for each ML model is shown in the plots of Figure 3; for most models, the most important features are the mean value of Mel coefficients 1, 2, 9 and in lesser proportion 3, 6, 12. Furthermore, there are features with a minimum effect, such as the mean value of Mel coefficients 4 and 11.

### 4.2. Performance Evaluation by CM

**Figure 4:**
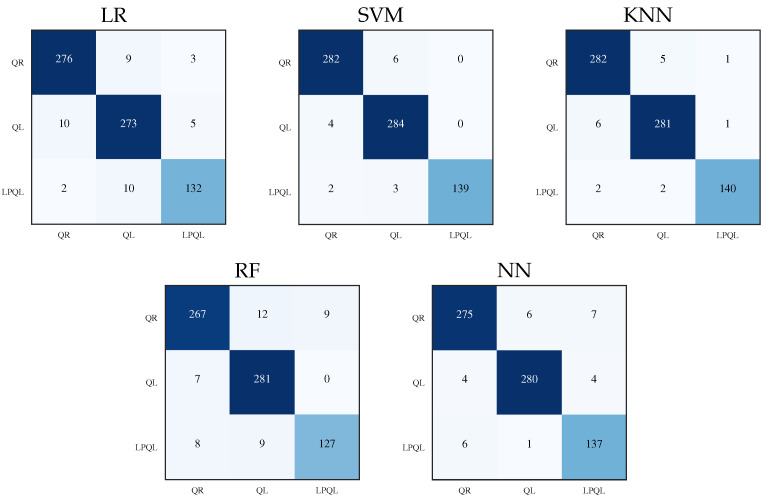
Confusion matrices summary for an eight-fold cross validation.

The CM and their performance metrics were computed in the process of eight-fold cross-validation; every fold consists of 90 samples; 36 samples for QR colonies, 36 samples for QL colonies, and 18 samples of LPQL colonies. The accumulated samples for the eight-fold cross-validation are 288 for QR colonies, 288 for QL colonies, and 144 for LPQL. The results of every fold are summarized in a single CM for each ML model, as shown in Figure 4. The elements in the main diagonal represent the TP values for their respective class. For QR colony classes, the TP value is the first cell of the matrix (1,1), the FN values correspond to cells (1,2) and (1,3), the FP values correspond to cells (2,1) and (3,1), and the TN values are the rest of the cells. For QL and LPQL classes, the corresponding TN, FN and FP values must be reassigned.

Analyzing the main diagonals in the confusion matrices, Figure 4, the SVM and KNN models achieved the highest correct classification values, the classifiers with the lower performance are LR and RF, followed by the NN model with moderate values. For a better assessment, the misclassification rate for each class and ML model is presented in Table 6, and the RF model presents the highest classification error; it is easy to notice that the best results were obtained with KNN and SVM models.

### 4.3. Performance Metrics Comparison

The performance metrics for each ML model are shown in Table 7, and the mean value for the eight-fold cross-validation procedure is shown beside the standard deviation (std). The lowest values of CM metrics were found with LR and RF models, and the std value reveals more variability in data. The best performance was achieved with SVM, KNN, and NN. The results of the KNN model are very consistent as it shows the std value.

**Figure 5:**
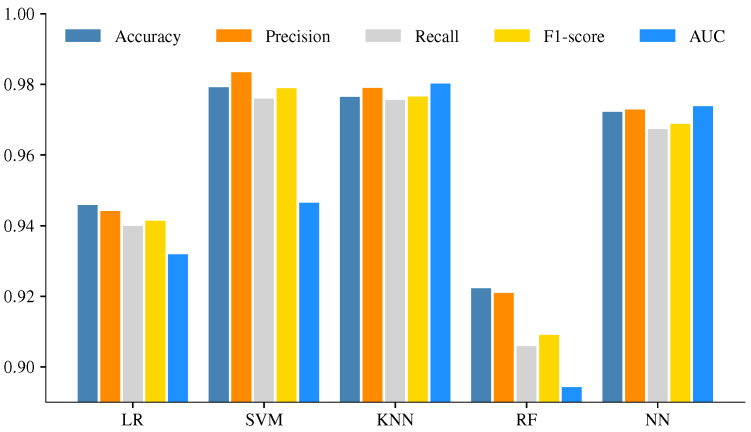
Performance metrics comparison of ML models.

For an easy way to compare the results, Figure 5 shows the mean value of the metrics. In this comparison, the results of SVM, KNN and NN are very similar; the lowest performance was obtained with RF, followed by LR. However, most of the values are above 90% and can be considered well-suited to discriminate the condition of the colonies.

**Figure 6:**
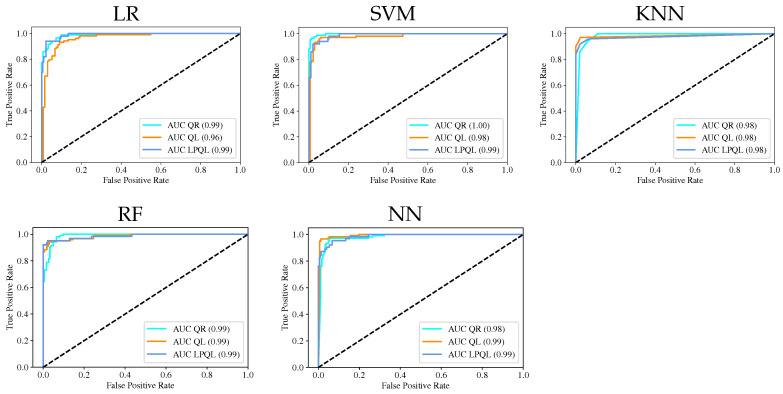
ROC curves for each ML model.

The ROC curves were obtained using the OvA methodology, Figure 6; in this case, the dataset was divided into 70% for the training set and 30% for the testing set. Three ROC curves were computed for every ML model, corresponding to every class in the problem; the AUC value is shown in the lower right corner. In all cases, the AUC values are above 0.95, with no significant differences, which means that all the ML models can discriminate between classes or colony states, and the most important is that all the classes were correctly identified.

### 4.4. Overfitting Verification by k-Folds Cross Validation

**Figure 7:**
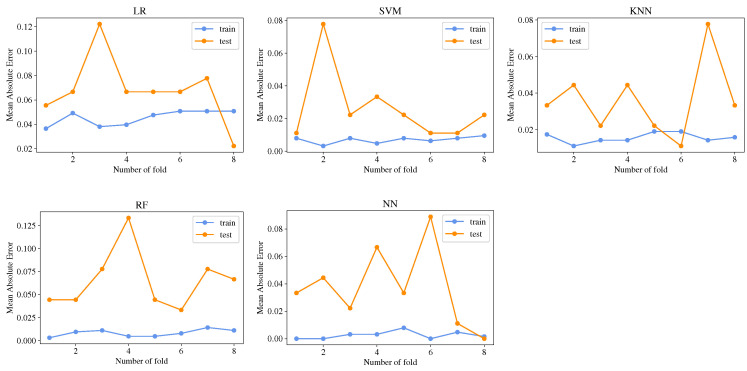
Overfitting verification in ML models by computing the mean absolute error in the k-fold cross validation.

Using the k-fold cross validation, the mean absolute error was calculated in every fold to verify overfitting in the ML models. In Figure 7 mean absolute error is plotted against the number of folds, and it can be noted that the training error is low (about 0.02) for all models except for LR. However, the test error presents more variations; the maximum error in RF and LR can be found in values around 0.12 and, for the rest of the models, around 0.08. Nevertheless, the plots do not present huge variations of error; therefore, it can be concluded that the models do not present overfitting.

### 4.5. Computational Cost

**Figure 8:**
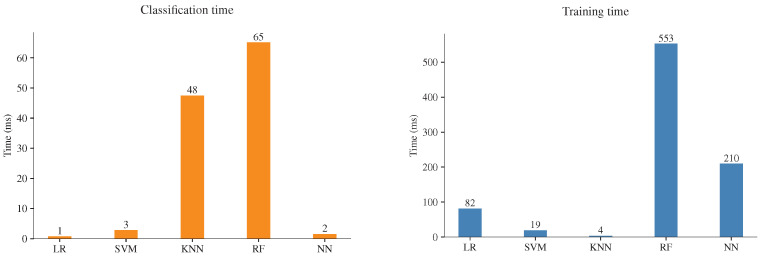
Training and classification time comparison.

Finally, as a form to evaluate the computational cost, the time required to classify the samples and train the models was measured by using the library *time* in Python. The training time is the time required to train an ML model; the classification time is the time required to classify the test dataset. The execution time considers the single line of code where training and classification take place, meaning that the feature extraction, preprocessing, and dataset splitting are out of the measurements. The training time was measured by using the 70% of the dataset (504 samples) and the classification time of the rest of the dataset (216 samples). As mentioned previously, the experiments of time execution were carried out on an RPi 3 computer; moreover, to take advantage of the device resources, the computer was run with no graphical desktop and no other application running at the same time. It is important to note that the implementation of ML models only uses one core of the processor of the four available. The experiments for every ML model were repeated ten times, and the results were summarized with the mean value, Figure 8. Most of the models are executed in very short times; this was achieved by computing the mean value of the MFCCs; without this step, this process takes more time on a regular PC. The results highlight significant differences; RF is the model that requires more training time, followed by NN and LR; on the other hand, SVM and KNN models require the shortest training times, which is essential if the model needs to be trained in the monitor system. This task is fast even in the slower ML model, but the increase of the dataset will be necessary to generalize between colonies and status health. At this point, the dataset size is limited due to the difficulty of obtaining new samples; however, increasing the dataset size will require more training time, and the execution time difference will be noticeable. The classification time is the most critical measure; this task will be performed several times to recognize the colony state and will be one of the most consuming resource processes in a monitor system. The results, Figure 8, show that RF and KNN are the models that require more time to classify the samples. In this experiment, RF is the model that requires significant training and classification time. Finally, the faster classification time is achieved by SVM and NN, followed by LR.

In summary, RF has the highest training and classification time, the lowest performance, and the highest classification error; therefore, RF is discarded as a classification model. On the other hand, considering the simplicity of the KNN model, the performance achieved is one of the best, with the lowest classification error; their metrics present high performance and low variability; the only shortcoming is the slow classification time. The LR model has the fastest classification time; however, a slightly low performance compared with the rest of the ML models. Finally, the models considered well suited for a monitor system are SVM and NN, with the faster classification time and highest performance metrics.

## 5. Conclusions

This study presents an extensive and detailed comparative analysis of the performance of five classification ML models for pattern recognition of colony states based on bee acoustics. The main goal of the analysis was to find an ML model with a balance between high performance and low computational cost. The results show the feasibility of implementing a classification task on a monitor system whose principal component is a single-board computer with low computational resources; the appropriate selection of the ML model is necessary to improve the system’s performance and extend the battery life.

The results show that all the models are efficient and produce excellent performance with a low classification error. When timing performance results are considered, the NN and SVM models highlight the faster training and classification time; therefore, NN and SVM are the most suitable for use on devices such RPi with low computational resources. Training the ML models in the device could be an interesting proposal when we are in real-life conditions; most of the apiaries are in remote places, and have the possibility to make immediate modifications and test the algorithms in the site could be an advantage. Furthermore, since the codes were written in Python, they can be easily exported and executed on any device with the python libraries. Moreover, considering the constant renovation of RPi hardware with superior characteristics, a newer RPi 4 with a superior processor can execute the codes in shorter times.

Even when the focus of this research work is not the methodology for prepossessing and feature extraction; the results show that the final dataset is compact and have enough information to discriminate the colony states; this was achieved by computing the mean value of the MFCCs, the efficiency of the classifiers evidences it. The above is one of the most critical factors and allows the possibility of implementing the complete methodology (sound recording, data preprocessing, feature extraction, and classification) in dedicated hardware.

In future work, a mandatory step that allows the generalization is to increase the dataset size and analyze more bee colonies in conditions that affect their acoustics. Furthermore, an analysis of the samples recording time will be conducted to reduce the computational complexity and space requirements and improve the prepossessing data step, which is one of the most demanding stages of the acoustic classification process. As long as the dataset size increases and the number of colony states to recognize, it could be necessary to implement more complex models, such as deep neural networks; an alternative is the Pytorch library, which offers GPU support and can take advantage of more advanced devices such as the Jetson Nano computer. Moreover, actual research trends aim to solve some inconveniences in CNN, and some proposes exist to reduce the time complexity to allow CNN for real-time applications [22]. Finally, understanding that the results obtained to date are laboratory experiments, the effectiveness of the ML models needs to be tested in real-life conditions; further investigations will be carried out in an apiary, and this is the next step in the development of the monitor system.

## Figures and Tables

**Table 1:** Estimation of the population size of the bee colonies, queenright colony (QR), queenless colony (QL), low-population queenless (LPQL).

	Colony 1	Colony 2	Colony 3	Colony 4	Colony 5
Thousand of bees	60	60	30	40	40
Colony states	QR	QL (removed)	LPQL	QL (removed)	QR

**Table 2:** MFCCs parameters.

Frequency Sampling	Frame Duration	Frame Shift	Preemphasis Coefficient	Window	Frequency Range for Filterbank
(Hz)	(ms)	(ms)			(Hz)
4000	25	10	0.94	Hamming	10–2000

**Table 3:** Metrics based on the CM.

Metrics	Formula
Acuracy	$\frac{\left(TP+TN\right)}{\left(TP+TN+FP+FN\right)}$
Precision	$\frac{TP}{\left(TP+FP\right)}$
Recall	$\frac{TP}{\left(TP+FN\right)}$
F1-score	$2\frac{Prec\;*\;Rec}{\left(Prec+Rec\right)}$
Misclasification	$\frac{\left(FP+FN\right)}{\left(TP+TN+FP+FN\right)}$

**Table 4:** Raspberry Pi 3 technical specification and setup.

Specifications	
Chipset	Broadcom BCM2837B0
Processor	Cortex-A53 64-bit SoC @ 1.4 GHz
Memory	1GB LPDDR2 SDRAM
Input power	5 V/2.5A DC
Operating system	Raspbian Buster v10
Data storage	32 GB micro SD card

**Table 5:** Hyperparameter of ML models estimated by random search.

LR	SVM	KNN	RF	NN
*LogisticRegression*	*SVC*	*KNeighborsClassifier*	*RandomForestClassifier*	*MLPClassifier*
Solver: *lbfgs*	Kernel: *rbf*	Weights: ‘distance’	Num estimators: 68	Solver: *lfbgs*
C value: 1.82	C value: 1.015	Neighbors: 30	Criterion: ‘entropy’	Activation: ‘relu’
Penalty: l2	Gamma: 0.065	Algorithm: ‘brute’	Boostrap: True	Alpha: 0.86
		*p* value: ‘manhattan distance’	Max depth: 10	Hidden layer sizes: (64, 33)

**Table 6:** Misclassification rate (%) per class for each CM.

Class	LR	SVM	KNN	RF	NN
QR	3.33	1.66	1.94	5	3.19
QL	4.72	1.8	1.94	3.88	2.08
LPQL	2.77	0.7	0.83	3.61	2.5
Accumulated	10.82	4.16	4.71	12.49	7.77

**Table 7:** Summary of comparative metrics in a eight-fold cross validation procedure.

Metrics	LR		SVM		KNN		RF		NN	
	Mean	Std	Mean	Std	Mean	Std	Mean	Std	Mean	Std
Accuracy	0.946	0.035	0.979	0.020	0.976	0.017	0.922	0.032	0.972	0.023
Precision	0.944	0.037	0.983	0.015	0.979	0.015	0.921	0.032	0.973	0.020
Recall	0.940	0.042	0.976	0.029	0.976	0.022	0.906	0.049	0.967	0.034
F1-score	0.941	0.040	0.979	0.023	0.977	0.018	0.909	0.042	0.969	0.028
AUC	0.932	0.051	0.946	0.041	0.980	0.020	0.894	0.069	0.974	0.033

## Data Availability

We made use of the publicly available dataset in https://data.mendeley.com/datasets/t9prmbmdfn, accessed on 9 September 2022.

## Abbreviations

The following abbreviations are used in this manuscript:

AUC	Area under the ROC curve
CM	Confusion Matrix
CNN	Convolutional Neural Networks
FFT	Fast Fourier Transform
FN	False Negative
FP	False Positive
HHT	Hilbert Huang Transform
HMM	Hidden Markov Models
KNN	K-Nearest Neighbors
LPQL	Low population queenless colonies
LR	Logistic Regression
MFCCs	Mel Frequency Cepstral Coefficients
ML	Machine Learning
NN	Neural Networks
OSBH	Open Source Beehive
OvA	One-vs-all
QL	Queenless Colonies
QR	Queenright Colonies
RF	Random Forest
TN	True Negative
TP	True Positive
